# Isolation and Characterization of a *Serratia rubidaea* from a Shallow Water Hydrothermal Vent

**DOI:** 10.3390/md21120599

**Published:** 2023-11-21

**Authors:** Ricardo F. S. Pereira, Maria J. Ferreira, M. Conceição Oliveira, Maria C. Serra, Carla C. C. R. de Carvalho

**Affiliations:** 1 Department of Bioengineering, iBB—Institute for Bioengineering and Biosciences, Instituto Superior Técnico, Universidade de Lisboa, Av. Rovisco Pais, 1049-001 Lisboa, Portugal; ricardofspereira@tecnico.ulisboa.pt; 2 Associate Laboratory i4HB, Institute for Health and Bioeconomy, Instituto Superior Técnico, Universidade de Lisboa, Av. Rovisco Pais, 1049-001 Lisboa, Portugal; 3 Centro de Química Estrutural, Institute of Molecular Sciences, Instituto Superior Técnico, Universidade de Lisboa, 1049-001 Lisboa, Portugal; m.joao.ferreira@tecnico.ulisboa.pt (M.J.F.); conceicao.oliveira@tecnico.ulisboa.pt (M.C.O.); 4 Área Departamental de Engenharia Química, Instituto Superior de Engenharia de Lisboa (ISEL), Rua Conselheiro Emídio Navarro, 1, 1959-007 Lisboa, Portugal; mcserra@deq.isel.ipl.pt

**Keywords:** marine bacteria, *Serratia rubidaea*, prodigiosin, upstream, downstream, bioprocess, NMR, DSC, UHR-QqTOF

## Abstract

Microbial life present in the marine environment has to be able to adapt to rapidly changing and often extreme conditions. This makes these organisms a putative source of commercially interesting compounds since adaptation provides different biochemical routes from those found in their terrestrial counterparts. In this work, the goal was the identification of a marine bacterium isolated from a sample taken at a shallow water hydrothermal vent and of its red product. Genomic, lipidomic, and biochemical approaches were used simultaneously, and the bacterium was identified as *Serratia rubidaea*. A high-throughput screening strategy was used to assess the best physico-chemical conditions permitting both cell growth and production of the red product. The fatty acid composition of the microbial cells was studied to assess adaptation at the lipid level under stressful conditions, whilst several state-of-the-art techniques, such as DSC, FTIR, NMR, and Ultra-High Resolution Qq-Time-of-Flight mass spectrometry, were used to characterize the structure of the pigment. We hypothesize that the pigment, which could be produced by the cells up to 62 °C, is prodigiosin linked to an aliphatic compound that acts as an anchor to keep it close to the cells in the marine environment.

## 1. Introduction

In the last decade, more attention has been given to the marine environment and its natural biological resources. The quest for interesting bioactive products from marine organisms has been stimulated due to the fact that most of these compounds have the potential to be developed industrially as pharmaceuticals, cosmetics, nutritional supplements, molecular probes, fine chemicals and agrochemicals, pigments, dyes, and photosensitizers [1,2,3,4,5,6]. These marine organisms, from sponges to viruses, developed adaptation strategies allowing them to survive and thrive in a myriad of hostile and extreme conditions, such as high and low temperatures (2–100 °C), high salinity (up to nearly saturation), and high pressure (>100 atm), and to survive attacks by predators and pathogens [7,8]. The ability of microorganisms to maintain biological functions under stress results from, e.g., changes in protein, sterol, hopanoid, carotenoid, and membrane lipid composition [9,10] and from the synthesis of primary and secondary metabolites [7].

The majority of the products found to be interesting, whose concentration is affected by the location where their producers prosper, are secondary metabolites, as they lack an apparent function during growth [7]. Among these metabolites, pigments have always attracted much attention since ancient times, such as tyrian purple. One pigment that is gaining much interest is prodigiosin, a red colour compound which belongs to the broader family of prodiginines, which are tripyrrolic red pigments presenting immunosuppressive and anticancer, antifungal, antibacterial, antiprotozoal, and antimalarial activities [11]. Prodigiosin is a lead compound of the 4-methoxypyrrolyldipyrrin family of natural products [12,13], and many synthetic derivatives of it or “prodigiosenes” are currently in phase I and II of clinical trials in the USA for solid tumours and hematologic malignancies but are not yet available on the market [13,14]. The exact mechanism(s) of in vivo anticancer activity needs further investigation. However, it has been proposed that one or more of the four following mechanisms may be occurring: (i) cytotoxicity including DNA damage; (ii) intracellular acidification; (iii) kinase modulation; and (iv) apoptosis induction [15,16]. Biologically, these structures are easy to produce due to the absence of harsh acidic conditions, necessary for the chemical decarboxylation of the pyrrole rings to give the desired multipyrrolic skeleton [13].

Prodiginines are produced mainly by *Serratia* spp. but may also be synthesized by actinomycetes and various marine bacteria [11,13,16,17]. These cells use biochemical routes in which monomeric pyrroles establish chemical bonds between them by enzymatic condensation, creating multipyrrolic compounds, controlled by a prodigiosin biosynthesis gene cluster (pig cluster) in *Serratia* species. From the ten species of *Serratia* that have been described, prodigiosin production has been mainly reported in *S. marcescens* strains and only in *S. plymuthica*, *S. rubidaea*, and *S. nematodiphila*, being responsible for the red-coloured colonies [18]. Most of the research in this field has involved improving production conditions or genetic features of *S. marcescens* strains. However, *S. marcescens* strains can be pathogens and cause nosocomial infections [19].

In the present study, a bacterium producing red colonies was isolated from a sample collected near a shallow water hydrothermal vent. Several techniques were used to identify the bacterial species and to characterize the pigment. Additionally, the conditions influencing its production were studied to find those leading to high yields. The adaptation strategies and advantages given to the bacterium by the ability to produce the red compound are also discussed.

## 2. Results and Discussion

### 2.1. Bacterial Identification

Initial identification was carried out using the Sherlock^®^ Microbial Identification System from MIDI, Inc. (Newark, DE, USA), which is based on the fatty acid profile of the strain [20,21]. Analysis following growth in TSA and BA plates for 24 ± 1 h suggested that the strain belongs to the Enterobacteriaceae family when compared to the species in the Sherlock library. The similarity indexes were the following: 0.659—*Escherichia coli* GC subgroup K-12 (confirm with other tests); 0.564—*Proteus vulgaris* (confirm with other tests); 0.548—*Serratia marcescens* (confirm with other tests).

The biochemical assays RapID^TM^ One and RapID^TM^ SS/u Systems (Remel^TM^, Thermo Scientific^TM^, Lenexa, KS, USA) were used to further help the identification of the bacterium. Using the ERIC^®^ database (http://www.remel.com/eric/, accessed on 11 December 2018 and confirmed on 10 March 2023), the results of the SS/u system identified the bacterium as *Serratia* spp., whilst the RapID^TM^ One system identified it as *S. rubidaea*.

**Figure 1:**
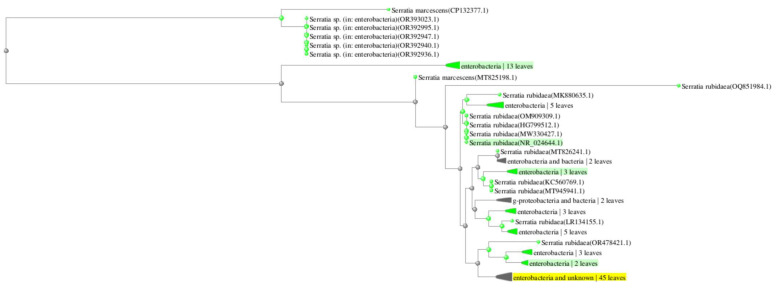
Molecular phylogenetic analysis with neighbour joining method. The tree represents a comparison between the 16S rRNA coding gene sequence of the new isolate, highlighted in yellow, and those of species in the BLAST database for a maximum sequence difference of 0.5. The taxonomic name and NCBI access number are indicated for each sequence.

Sequencing of the 16S rRNA of the bacterium and BLAST analysis of the consensus sequence showed that the isolated strain presents over 99.7% similarity to sequences of several *S. rubidaea* listed (Figure 1).

*S. marcescens* strains can be pathogens and cause nosocomial infections, contrarily to *S. rubidaea* strains, which have rarely caused infections in humans [19]. Additionally, *S. marcescens* strains usually require long incubation periods to synthesize prodigiosin and cannot produce the pigment above 36–37 °C due to the denaturation of heat-sensitive enzymes involved in the biosynthesis pathway [22] and/or due to the temperature regulation of prodigiosin production at the transcriptional level [18]. Due to the particular environmental conditions of the sampling site of this study, and the lack of suitable bioprocesses using *S. rubidaea* for the production of prodigiosin, the growth and production conditions of the isolated strain were further studied.

### 2.2. Assessing Growth Conditions

Since the strain was isolated from a marine sample, the effect of salt was initially assessed in 24-well MTPs using sea salts, a modified LB medium (RP) [20], and TB [23]. The media were also tested with sodium phosphates replacing the regular potassium phosphates and with 30 g/L NaCl replacing the latter. The production of a red pigment was only observed when NaCl was present. Prodigiosin production in *S. marcescens* is usually inhibited at 30 g/L NaCl [18], but such concentration favoured the growth of the isolated *S. rubidaea*. Assays on mineral medium without a carbon source also showed that the cells are not autotrophic.

Different complex media, containing peptone, tryptone, or yeast extract, were used to determine the best conditions to grow the isolated *S. rubidaea* strain by submerged fermentation. Since, as mentioned, a red pigment was only produced in the presence of salt, all tested media were supplemented with NaCl up to a concentration of 30 g/L of NaCl. The exceptions were media F2 and BG-11, which were not supplemented with NaCl. However, these media were supplemented with 5 g/L of meat peptone and 1 g/L of yeast extract (F2S and BG-11S).

**Figure 2:**
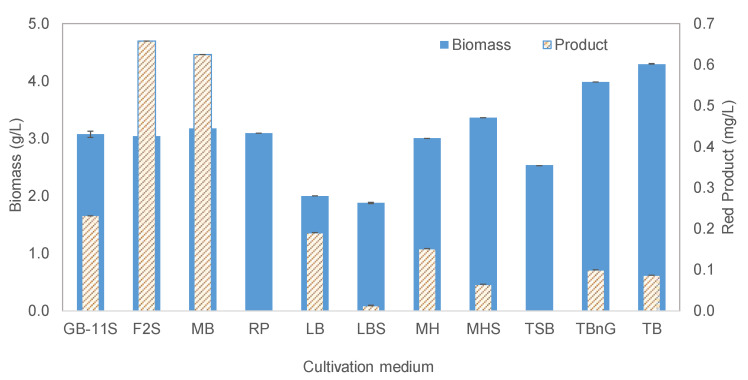
Biomass and red product concentration produced by *S. rubidaea* cells grown on different liquid media at 30 °C and 150 rpm. Abbreviations: GB-11S (GB-11 + 5 g/L meat peptone + 1 g/L yeast extract), F2S (F2 + 5 g/L meat peptone + 1 g/L yeast extract), LBS (LB + 3% NaCl), MHS (MH + 3% NaCl), TBnG (TB without glycerol). Values are represented as average ± standard deviation.

The concentration of biomass and of a deep red compound depended on the cultivation media (Figure 2). After a preliminary UV-VIS characterization, the red product was identified as a prodiginine, which are tripyrrolic dark red compounds produced by several species of the genus *Serratia* as secondary metabolites [24].

Both marine-based media, F2S and MB, allowed the highest product concentrations, which reached 0.66 and 0.62 mg/L, respectively (Figure 2). Since F2 is a seawater medium for marine algae, especially diatoms, it contains vitamins which are thermolabile and may decompose during autoclaving. This may hamper its utilization at an industrial scale, and thus MB was selected for further studies.

#### 2.2.1. Effect of Nutrient Concentration

When compared to commercial cultivation media, natural seawater has a much lower nutrient concentration, which may hamper marine bacterial growth at levels required for biotechnological applications [1,25]. Thus, the threshold concentration of nutrients under which the marine *S. rubidaea* strain was unable to grow was studied. According to Jannasch (1967), who studied the effect of extremely low concentrations on the growth of marine bacteria in a chemostat, two types of species of heterotrophic bacteria can be distinguished under depleted nutrient conditions: one type adapts to the marine environment by its ability to grow at low substrate concentrations; the other remains inactive but viable in natural seawater [25]. Additionally, in the ocean, oligotrophic bacteria grow faster than their copiotrophic counterparts, except when the latter are in the presence of favourable conditions [26]. In this case, copiotrophic bacteria can reach maximum growth rates much higher than oligotrophic bacteria.

**Figure 3:**
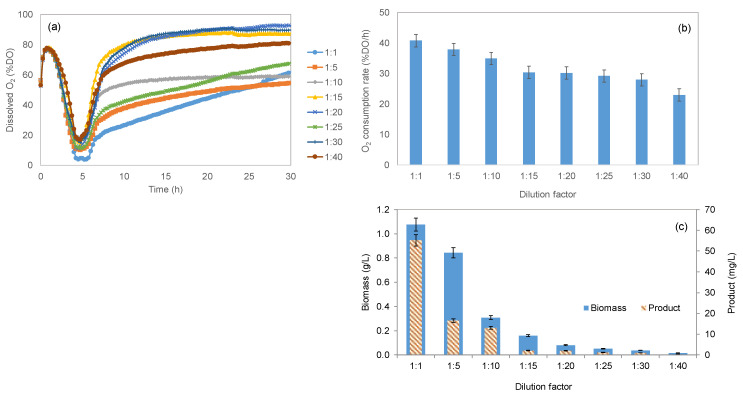
Effect of nutrient depletion on *S. rubidaea* growth: oxygen profile measured in real time during growth (**a**); oxygen consumption rate (**b**); and (**c**) biomass and product concentrations. The cells were grown for 30 h in MB media diluted up to 40 times at 30 °C and 150 rpm. Values are represented as average ± standard deviation, except in (**a**) for clarity.

To monitor the growth of the *S. rubidaea* strain under nutrient-depleted conditions, the dissolved oxygen was measured, in real time, using 24-well plates with an integrated fluorescence sensor spot. The dissolved oxygen may be used as a proxy for bacterial cell growth [27]. The exponential growth phase could be easily identified by a fast oxygen depletion in the cultivation medium (Figure 3a). Once the cells reached the stationary phase, an increase in dissolved oxygen could also be observed. The specific oxygen consumption rate decreased with the increasing dilution factor of the cultivation medium: a faster decrease (corresponding to 10.4% DO/h) was observed when the dilution factor increased to 1:15 than when the dilution factor increased from 1:15 to 1:40 (corresponding to 7.4% DO/h; Figure 3b). An exponential decrease both in attained biomass concentration (R^2^ = 0.97) and product concentration (R^2^ = 0.93) with increasing dilution factor was observed (Figure 3c). The *S. rubidaea* were therefore able to grow under low nutrient concentrations although at a low rate and reaching low concentrations of both cells and product. Since the sample from which the bacterium was isolated from was collected near a thermal spring in a marine natural pool, the mixture of sea and thermal water could in fact result in a very low nutrient concentration, contributing to the adaptation abilities of this bacterium.

#### 2.2.2. Effect of Temperature

As mentioned, the sampling site was near a shallow water thermal vent in an intertidal zone. The temperature of the thermal water is ca. 60 °C, resulting from ca. 50% seawater (pH 8) mixing with acid brackish water (pH < 5) which is at ca. 100 °C [28]. To simulate the conditions that the bacteria in this environment might endure, the cells were grown in shaken flasks in MB at the following temperatures: 15 °C and 20 °C to simulate high tide (ocean waves enter the pool); 30 °C to simulate low tide; and 62 °C, since this was the temperature referred by local authorities near the water spring on the sampling day. Since the bacterium belongs to the Enterobacteriaceae group, growth was also promoted at 37 °C.

**Figure 4:**
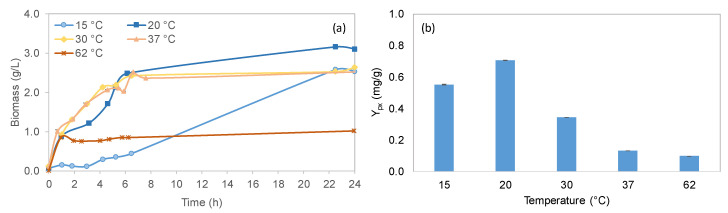
Influence of temperature on marine *S. rubidaea* growth and production on MB. (**a**) Growth curves observed for 24 h; (**b**) product from biomass yields. Values are represented as average ± standard deviation, except in (**a**) for clarity.

The studied *S. rubidaea* strains grew faster in MB at 30 and 37 °C than at 15 and 62 °C (Figure 4a). At a low temperature, the cells grew slowly but could attain the same concentration that was observed for cells growing at 30 and 37 °C after 24 h. On the contrary, cells growing at 62 °C attained a concentration of 0.85 g/L in the first 1.8 h, but no additional increase in cell number was observed.

Regarding the production of the red product, the highest product from biomass yield was observed at 20 °C, reaching 0.7 mg/g (Figure 4b). At 37 and 62 °C, *S. rubidaea* cells significantly decreased the production of the red product. It has been previously shown that *S. marcescens* strains cannot produce the pigment above 36–37 °C due to the denaturation of heat-sensitive enzymes involved in the biosynthesis pathway [22] and/or due to the temperature regulation of prodigiosin production at the transcriptional level [18]. The production of the red pigment prodigiosin in *S. marcescens* is thermoregulated at transcriptional level probably by the HexS transcription factor [29], and in the strain *S. marcescens* JNB 5-1, it was found that the two-component regulatory system *cpx* is regulated by temperature and inhibits prodigiosin production when the temperature increases from 30 to 37 °C [30]. The *S. rubidaea* isolated in the present study can produce prodigiosin at least up to 62 °C.

The bacterium showed a significant temperature window for growth, spanning at least 47 °C. This plasticity towards temperature, together with that showed with media with different salinities and nutrients, indicates that this strain is well adapted to continuously changing environmental conditions. Such conditions are observed in intertidal zones such as the one from which this *S. rubidaea* strain was isolated. Bacteria from extreme environments, such as *Mycobacterium* strains isolated from samples collected in Yellowstone and Glacier National Parks, have also been found to be able to grow at temperature values > 60 °C and under osmotic stress [31]. In this case, the *Mycobacterium* cells were able to adapt the fatty acid composition of their cellular membranes as a mechanism of adaptation.

**Figure 5:**
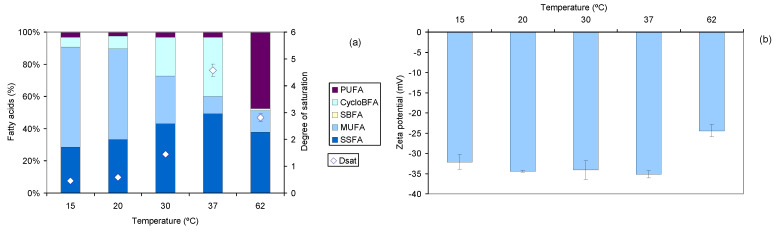
Influence of temperature on the cell envelope of *S. rubidaea*: (**a**) fatty acid profile (bars) and degree of saturation (diamonds); (**b**) zeta potential. Fatty acids: SSFA—saturated straight; MUFA—mono-unsaturated; SBFA—saturated branched; CycloBFA—saturated cyclopropyl-branched; PUFA—polyunsaturated; Dsat—degree of saturation. Values are represented as average ± standard deviation.

In the present study, the *S. rubidaea* cells also changed the fatty acid (FA) profile of their cellular membranes as the temperature increased when they were growing in MB (Figure 5). As temperatures rise, many FAs reach phase transition, resulting in the melting of 50% of the hydrocarbon chains and in the observation of both a liquid crystalline phase and a rigid gel phase [21,32]. While in the gel phase the phospholipid hydrocarbon chains are closely ordered, in the liquid crystalline phase disorder occurs and acyl chains may flap around [33]. Under these conditions, bacterial cells have to counteract this effect by producing fatty acids with higher melting points, such as long-chain saturated straight FAs, by changing the degree of saturation of the FAs of the membrane and/or by changing the conformation of the FAs [21,32,34].

*S. rubidaea* cells increased the content of saturated straight FAs with increasing temperature up to 37 °C while concomitantly decreasing the content of mono-unsaturated FAs (Figure 5a). This resulted in a 10-fold increase in the degree of saturation of the lipids of the cellular membrane when the cells grew at 37 °C compared to 15 °C (Figure 5a), indicating that the cells were able to counteract the fluidizing effect of higher temperatures. The saturated straight FA presenting the highest content was 16:0, whilst the main mono-unsaturated FAs in *S. rubidaea* were 16:1 ω7c and 18:1 ω7c (Table 1). Additionally, the cells increased by ca. 6-fold the content of saturated cyclopropyl-branched FAs (Figure 5a), mainly by increasing the content of 17:0 *cyclo* ω7c. These FAs reduce the fluidity of the cellular membrane by influencing lipid packing and could enhance the stability of the membrane at high temperatures as they are, in general, more ordered than the corresponding unsaturated chained FAs [21,35].

The most surprising change was, however, the production of large amounts of polyunsaturated fatty acids (PUFAs), reaching ca. 47%, when the cells grew at 62 °C (Figure 5a). The main PUFAs observed were 18:3 ω6c, 15:3 ω3c, and 16:4 ω3c (Table 1). Until the 1990s, it was considered that bacteria were unable to produce PUFAs, except some cyanobacteria, but it is now accepted that they are produced by many marine bacterial species, and by some species isolated from soil samples [34,36,37]. The synthesis of PUFAs has been observed during, e.g., the adaptation of *Rhodococcus erythropolis* cells to extreme conditions [34], and they were found to be fundamental for the rapid adaptation of these cells to salt-induced stress [38]. The presence of PUFAs has been linked to the maintenance of membrane fluidity in deep-sea prokaryotes to counteract the effect of high pressure and low temperature [21], but in *R. erythropolis* cells, they were apparently responsible for decreasing the number of negatively charged groups in ion channels, allowing the repellence of NaCl during osmotic stress [38]. In fact, it has been shown that PUFAs, such as omega-3 FA, may have very small or no effects on the fluidity of lipid bilayers [39]. In *S. rubidaea*, they were produced in large quantities only when the cells grew at 62 °C, which would already contribute to a fluidization of the membrane, and thus the role of these FAs should also be other than contributing to an increased fluidity of the membrane.

When the zeta potential, which is an indirect measure of cell surface charge, was studied in *S. rubidaea*, it could be observed that cells growing at 62 °C had a 24% decrease in their zeta potential in comparison to those growing at 15 °C, and a 28% decrease in comparison to those growing at 30 °C (Figure 5b). For most bacteria, the net surface charge is negative and is balanced by oppositely charged counter ions present in the surrounding medium [40]. In the present study, the cells presented more negative zeta potential values when grown at 20–37 °C (average of −34.6 mV) and less negative values for 62 °C. This could indicate that the PUFAs also contributed to a decrease in the number of negatively charged groups at the surface of the cells.

#### 2.2.3. Effect of pH

Bacterial evolution led to the development of several strategies allowing the cells to maintain a neutral intracellular pH regardless of the pH of the environment. The cells depend on pH homeostasis due to the distinct ranges of pH optimal functionality of their proteins [41,42]. Marine bacteria are exposed to pH gradients in their environment due to seasonal and diurnal variations and during phytoplankton blooms [43], and their ability to maintain the correct pH is paramount for their survival.

**Figure 6:**
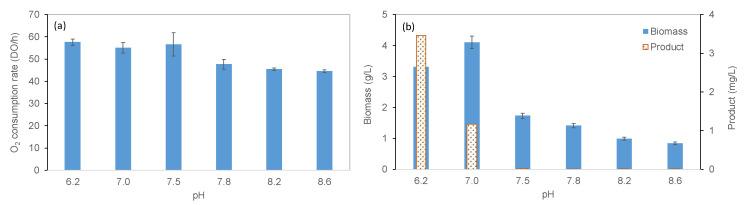
Effect of pH on oxygen consumption rate (**a**) and biomass and product concentration (**b**) during cultivation of *S. rubidaea*.

In the assays, the media pH was changed from 6.2–6.5 to 8.6–9.0. These include the values around which the majority of bacterial cells can grow and the pH normally measured in seawater. The assays were run at 30 °C since this allowed the fastest growth rate together with 37 °C but a higher productivity. A real-time system was used to assess the growth of the strain at different pH values, both by dissolved oxygen concentration and pH monitoring. Oxygen consumption rates by *S. rubidaea* decreased, in general, with increasing pH values (Figure 6a). The concentration of biomass and product were highest at pH 7.0 and 6.2, respectively (Figure 6b).

Curiously, the pH influenced the composition of FAs of the cellular membrane of the cells by inducing a reduction in the content of saturated cyclopropyl-branched FAs: from 28.1% at pH 6.2 to 15.2% at pH 8.6. In fact, the degree of saturation observed was on average 0.98 ± 0.14 for the tested pH values. The low degree of changes observed at the FA composition level may suggest that these cells are well adapted to pH fluctuations, not requiring the expenditure of energy necessary to change their lipid composition.

### 2.3. Product Identification and Characterization

**Figure 7:**
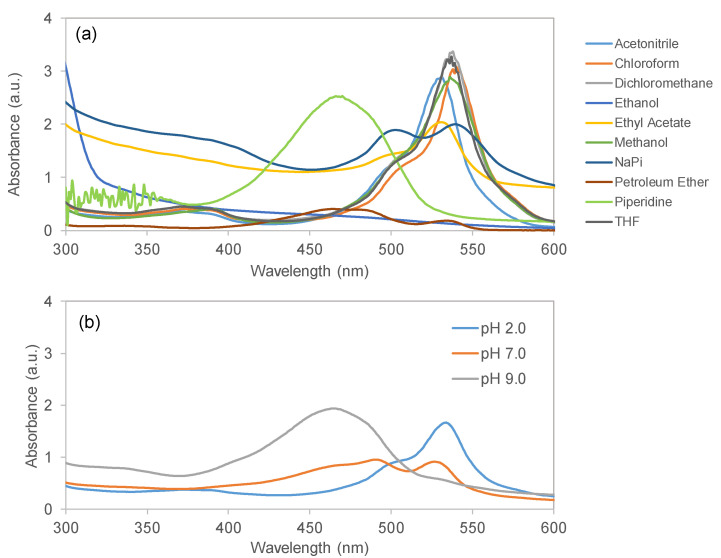
UV-VIS spectra of the red pigment produced by *S. rubidaea*. The pigment was dissolved in different organic and inorganic solvents (**a**). Effect of pH on the spectra of the product extracted with ethyl acetate (**b**).

Different solvents were tested to assess which were the most proper to extract the product from the biomass. For most of the solvents, the UV-VIS spectra of the product presented a maximum of absorbance at 535 nm but also a peak at 370 nm (Figure 7a). By comparison with the literature, the latter was presumed to be the Soret band [44]. The only changes to this profile were observed with piperidine, where the product presented the highest absorbance peak at 466 nm, and in phosphate buffer (NaPi), where the product showed two absorbance peaks at 500 nm and 535 nm (Figure 7a). Although both organic and inorganic solvents could extract the product from the cells, the organic solvents allowed the highest recoveries.

Following extraction from the cells, thin-layer chromatography (TLC) run in one dimension and as 2D, using dichloromethane as the eluent in one direction and methanol in the 90° direction, showed a single band in both cases. Its R_f_ value was 0.82.

Since this work was being conducted with a *S. rubidaea* strain, the product was hypothesized to be prodigiosin or a prodigiosin derivative. The information about prodigiosin is vast, but most papers report the production by *S. marcescens* [24,45]. One of the tests performed in the present work, to assess if the compound was similar to those described in the literature, was to compare the absorbance spectra when the product was placed at different pH values (Figure 7b).

By changing the pH of the solution containing the product, a bathochromic shift occurs in the peak observed at 535 nm under acidic conditions. At a neutral pH, a band with two peaks appears: one peak remains at 535 nm, but part of the product absorbance shifts to 491 nm. At a basic pH, the highest peak changes from 535 nm to 464 nm. By changing the pH to 2, 7, and 9, the product’s colour also changed between pink, red, and yellow, respectively. The results are similar to the ones reported in the literature [46,47], further suggesting a prodigiosin-like product.

**Figure 8:**
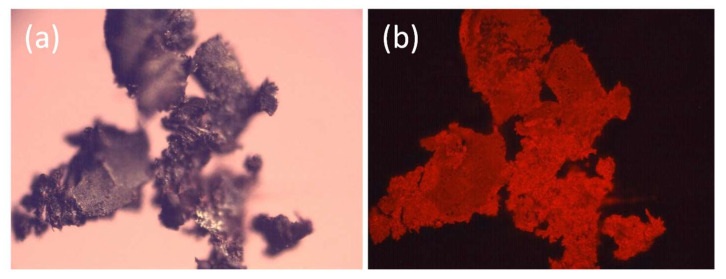
Appearance of the red product observed with microscopy under visible (**a**) and fluorescent (**b**) light. Magnification: 300×.

Small solid particles could be obtained with the evaporation of ethyl acetate, which could be observed with microscopy under visible and fluorescent light (Figure 8). The partially solid product was further analysed by fluorescence spectroscopy. This technique is considered as a primarily research tool in biochemistry, biophysics, biotechnology, flow cytometry, and medical diagnostics [48]. The compound presented a maximum excitation at 535 nm and a maximum emission at 560, regardless of the solvent used (e.g., methanol and ethyl acetate). This is also characteristic of prodigenine compounds such as prodigiosin [49].

**Figure 9:**
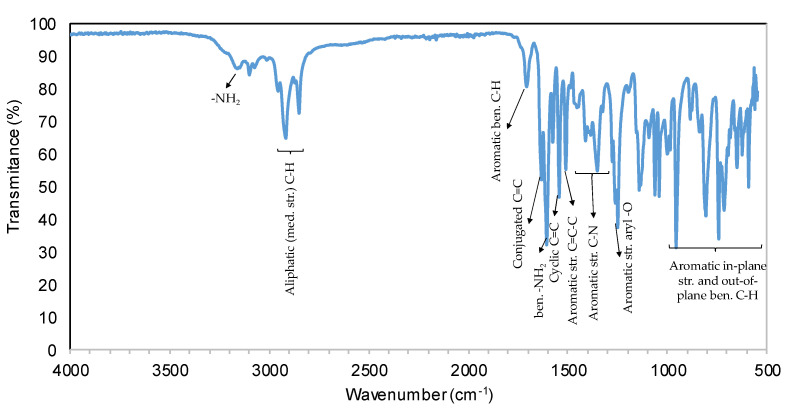
FTIR-ATR analysis of the powder of the dried purified product of *S. rubidaea*. Abbreviations: med = medium; str = stretch; ben = bending.

The red product was also analysed by Fourier-transform infrared spectroscopy—attenuated total reflectance (FTIR-ATR), which enables a rapid, non-destructive, reagentless, and high-throughput analysis of a diverse range of sample types [50,51] and requires a small amount of sample [52,53]. For metabolic compounds, FTIR is a valuable fingerprinting tool because it can analyse them simultaneously while requiring minimal sample preparation. In Figure 9, the FTIR spectrum of the dried purified compound is shown.

According to the spectrum, the product has the following characteristic peaks: a weak broad absorption at 3160 cm^−1^ corresponding to the N-H bonds of secondary amines; two peaks at 2920 and 2850 cm^−1^ corresponding to the medium stretching of C-H in alkenes and alkanes, respectively; a weak C-H bending at 1710 cm^−1^ typical of aromatic compounds; three peaks at 1630, 1580, and 1610 cm^−1^ corresponding to the C=C stretching of conjugated alkenes and cyclic alkenes and the N-H bending of amines, respectively; a weak broad peak at 1510 cm^−1^ corresponding to the C=C-C stretch of aromatic rings; two weak peaks at 1350 and 1280 cm^−1^ and a sharp peak at 1140 cm^−1^ that correspond to the C-N stretch of an aromatic secondary amine; a peak at 1250 cm^−1^ corresponding to the aryl -O stretch of aromatic ethers; and from 950 to 687 cm^−1^, there are several peaks that correspond to the out-of-plane bends of aromatic C-H bonds. These values are similar to those described for prodigiosin in the published literature [54,55,56].

According to previously published papers related to the purification of prodigiosin from *S. marcenses* [45,57,58,59,60], the final step involves the crystallization of the product. However, how this transition state occurs is variable according to the literature, and it is also described to occur within a variable timeframe, ranging from minutes to days, and at different temperatures, from −20 °C to room temperature. In the present study, regardless of the techniques tested, the obtained product never crystalized into a stable structure and remained in an amorphous state.

**Figure 10:**
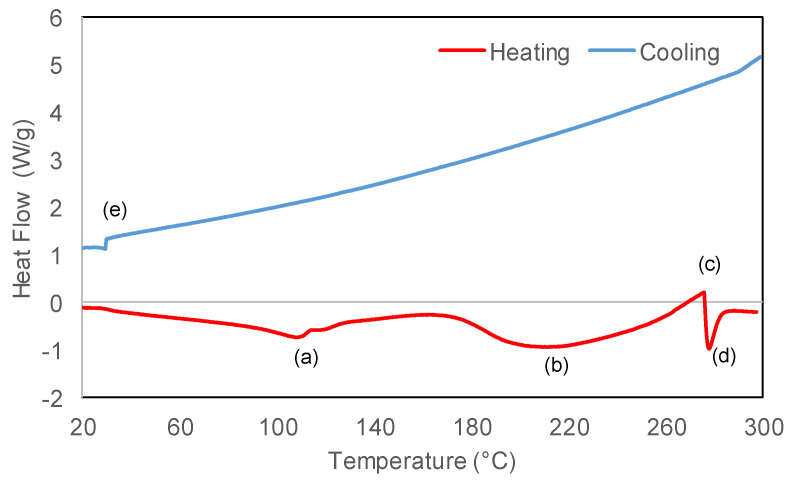
DSC heating and cooling curves of the purified red product.

Nunes et al. [61] showed that an amorphous material that can transit into a crystalized structure presents a crystallization exotherm in the DSC thermogram. However, the analysis performed by Ponjavic et al. [14] of a pure prodigiosin showed only a melting temperature (T_m_) at 201.4 °C. The amorphous state of our product suggested the use of DSC to determine its thermotropic properties. The thermogram of the red product of *S. rubidaea* presented the following features: (a) a glass transition point (T_G_) at 110.2°C, where a ladder step is present; (b) a T_m_ at 210.4 °C; (c) the crystallization exotherm at 277.6 °C; and (d) a sharp peak at 279.9 °C of a second T_m_ (Figure 10). The calculated first and second derivatives were utilized to determine exactly the key signal characteristics, such as the minima, maxima, and end and start points of thermal events. The T_G_ shows the product transition from dried powder to a more viscous state, whilst the presence of a second step may indicate either the presence of a polymorphism of the product or a contaminant linked to it. The 10 °C difference at point (b) in relation to previously published data [14] and the width of this peak suggest not only the presence of a contaminant but also the degradation of both products. The (c) and (d) points are more closely related to a polymer, thus indicating that the product is completed degraded at this temperature. With the cooling curve, it is possible to observe that only a sudden change in specific heat capacity occurs at (e), when the solidification of the amorphous form occurs at 29.58 °C.

Mass spectrometry (MS) offers good sensitivity and selectivity for the characterization of single compounds and metabolites. Metabolite profiles of biological origin have been commonly measured for several decades using gas chromatography– and liquid chromatography–mass spectrometry (GC-MS and LC-MS) [51]. While GC-MS may be used for volatile molecules, often requiring derivatization steps for the analysis of certain molecules such as lipids, LC-MS has the advantage of allowing the analysis of compounds in solution. The Ultra-High Resolution Quadrupole-Time-of-Flight (QqTOF) system used in this study allows for the high-resolution analysis of molecules even in complex mixtures.

**Figure 11:**
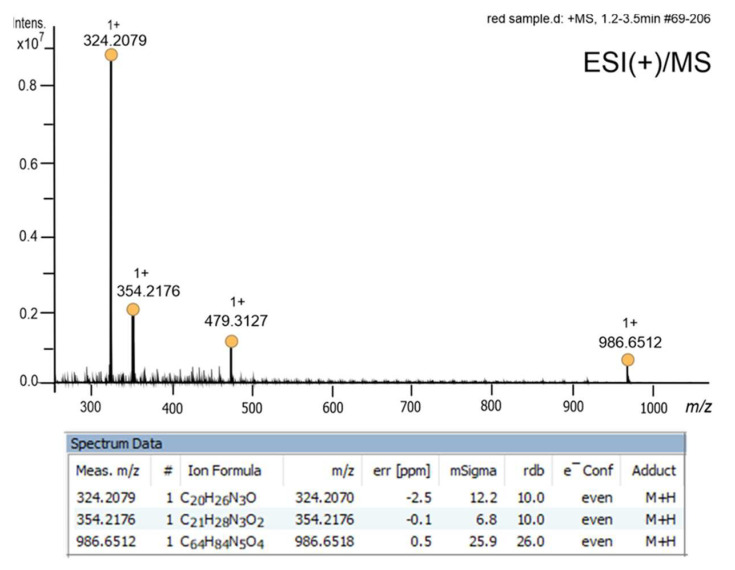
High-resolution full-scan mass spectrum in the ESI positive mode of the purified *S. rubidaea* product, and accurate mass measurement values for the protonated molecules of the prodigiosin-type structures identified in the spectrum.

**Figure 12:**
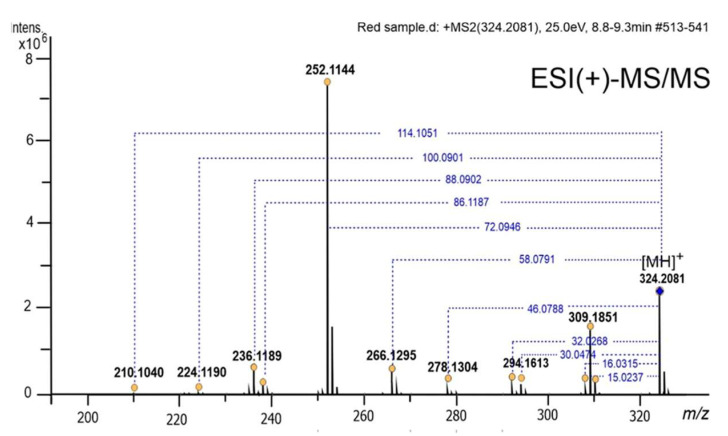
High-resolution tandem mass spectrum in the ESI positive mode for the precursor ion *m*/*z* 324.2081 assigned to an [C_20_H_26_N_3_O]^+^ structure.

**Scheme 1:**
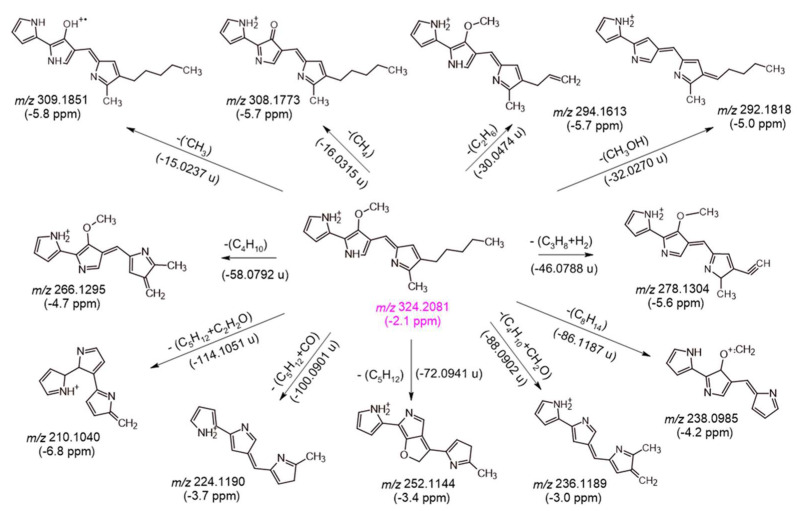
Proposed fragmentation path for the precursor ion *m*/*z* 324.2081 assigned to a prodigiosin-type molecule.

In the present study, the full-scan mass spectrum of the extract of the red product, in the ESI positive mode, showed as a base peak a signal attributed to a protonated molecule with *m*/*z* 324.2079, assigned to a [(C_20_H_26_N_3_O)^+^(Δ—2.5 ppm; mSigma 12.2)] structure. This result indicates that the red product must be a type of prodigiosin molecule. Three other peaks with very low intensity were also present: a peak with *m*/*z* 354.2176, which can be attributed to a rearrangement of prodigiosin with a CH_2_O group, a peak with *m*/*z* 479.3127 not identified as related to any prodigiosin rearrangement; and a protonated molecule with *m*/*z* 986.6512, which could be attributed to a rearrangement between a prodigiosin molecule and a tambjamine-type molecule. (Figure 11). To support that the main compound present in the red extract has a prodigiosin-type structure, the precursor ion *m*/*z* 324.2081 was isolated, and a collision-induced dissociation (CID) analysis with nitrogen was performed. The high-resolution tandem mass spectrum is presented in Figure 12. The fragmentation mechanism for the protonated molecule of prodigiosin is proposed in Scheme 1.

**Figure 13:**
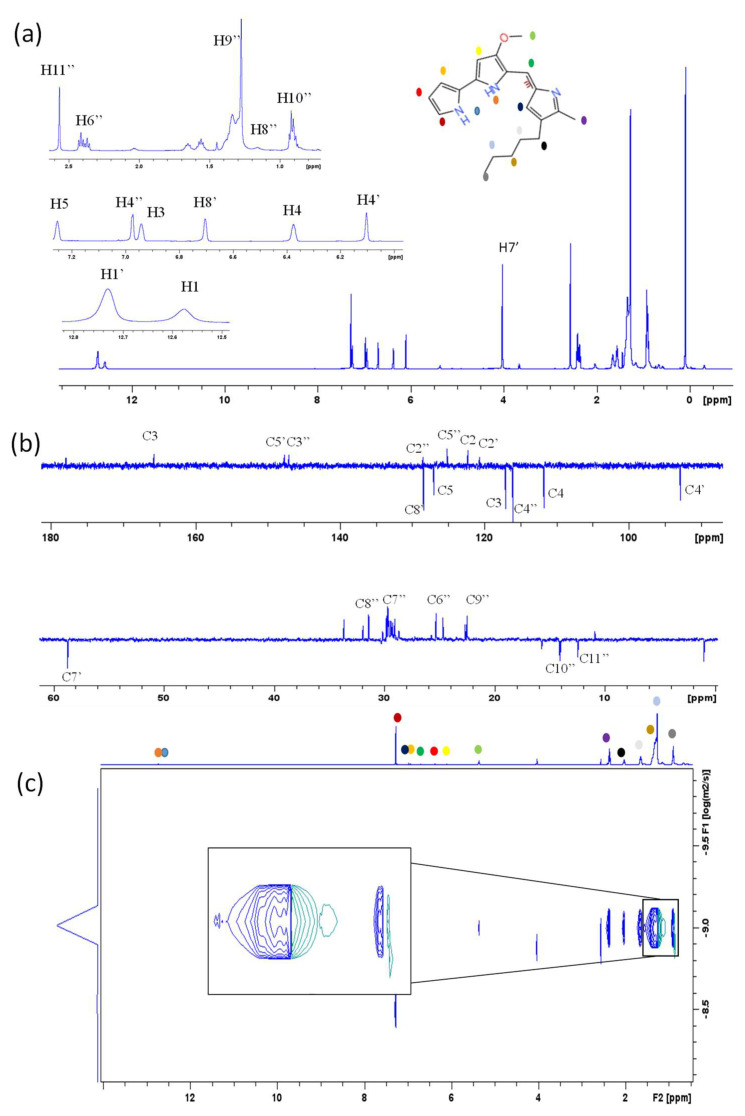
The 1D and 2D NMR analyses of the products of *S. rubidaea*. (**a**) ^1^H-NMR, (**b**) ^13^C-NMR, and (**c**) ^1^H-^1^H DOSY with the two molecules present identified in accordance with the bonded protons. All spectra were conducted in CDCl_3_, with its protons identified.

NMR was also used for the characterization of the structure of the product of *S. rubidaea*. NMR is a non-destructive and non-invasive technique that allows for the structural and conformational analysis of complex molecules and quantitative analysis of complex mixtures [51,62]. The ^1^H and ^13^C{1H} NMR data are consistent with the proposed structure suggested in [63] as we can identify the two N-H protons and all aromatic protons and carbons, as well as the signals assigned to the double bond and to the aliphatic chain (Figure 13). The signal assignment was aided by several 2D experiments, and the NMR results were complemented with the elemental analysis of the product. All NMR signals integrated correctly with the exception of those assigned to the aliphatic chain. Besides the red product, *S. rubidaea* also co-produced a white solid product that we hypothesize to be a polymer and that has signals in that region. This is consistent with the broad signals visible in the spectrum. We suspect that there is a close interaction between the molecule and the polymer as DOSY experiments (Figure 13c) show two different species but with very similar diffusion coefficients, suggesting that there is an equilibrium in solution, as depicted in Equation (1). This equilibrium may explain the failed attempts to obtain crystals of prodigiosin.

(1) $${\left(Pr\right)}_{red}+Polymer⇄\left(Pr\right)Polymer$$

Since the cells are from a marine environment, it could be hypothesized that the cells use a polymer-like compound as an anchor for the prodigiosin. In low-nutrient environments, the amount of energy necessary to produce a compound such as prodigiosin is valuable. If the cells simply released the compound extracellularly, it could be lost within a short period of time due to, e.g., currents and waves. The presence of an anchor-like aliphatic compound could maintain prodigiosin close to the cells. It has been found that the pigmentation of *S. marcescens* cells is correlated with an increased rate of ATP production during the lag phase of growth, and prodigiosin production may provide a growth advantage to cells in comparison to non-pigmented counterparts [64].

In Table S1, in the Supplementary Materials, all the assigned ^1^H, ^13^C chemical shifts, proton integration, and NOE observed in the 1D and 2D spectra are shown.

## 3. Materials and Methods

### 3.1. Sample Collection and Bacterial Isolation

Samples of water and sediments were collected on the island of São Miguel, the Azores, from 1 December to 3 December 2015, as described by Rodrigues et al. [1]. The *Serratia* strain used in this work was isolated from a sample collected near a shallow water thermal vent, on Piscina Natural da Ferraria, São Miguel, Portugal. The water of the thermal spring was at ca. 62 °C, but the mixture of sea and thermal water was at 28 °C. Table 2 shows the GPS coordinates and physical parameters measured in situ.

The bacterium was isolated in tryptic soy agar (TSA) and selected for its bright red colour. After identification as *Serratia*, it was stored at 4 °C in cryovials containing 1 mL of TSA at –80 °C in 20% glycerol and deposited and maintained at IST. The isolated colony was inoculated in new TSA and Marine Agar plates (MA; Conda) and incubated at 30 °C for 24 h.

### 3.2. Bacterial Identification

The bacterium was initially identified by its lipid profile using the Sherlock^®^ Microbial ID System from MIDI, Inc. (Newark, DE, USA). Briefly, the cells were grown on both tryptic soy agar (TSA) and blood agar (BA) plates (both from Fluka) at 30 °C for 24 ± 1 h. After harvesting the cells, their fatty acids (FAs) were extracted and simultaneously methylated to fatty acid methyl esters (FAMEs) using the Instant FAME^TM^ method from MIDI, as previously described [1,20]. FAMEs were analysed with gas chromatography on an Agilent Technologies 6890 N gas chromatograph (Agilent, Santa Clara, CA, USA), with a flame detector and a 7683 B series injector, using a 25 m Agilent J&W Ultra 2 capillary column (also from Agilent). Strain identification was performed by comparing the FAME profile of the cells with those in the database of the Sherlock^®^ software package (version 6.2) using the ITSA and IBA methods.

The identification was also carried out using the biochemical assays RapID^TM^ One and RapID^TM^ SS/u Systems (Remel^TM^, Thermo Fisher^TM^, Waltham, MA, USA) and with 16S rRNA sequencing. The extraction of DNA from colonies was carried out using the DNeasy Powerwater Kit (Quiagen GmbH, Hilden, Germany). The purification of DNA, PCR amplification of the segment of the 16S rRNA gene containing the variable regions V1-V9, and generation of the consensus sequence was conducted by Stab Vida (Caparica, Portugal). BLAST analysis of the consensus sequence was performed using the National Center for Biotechnology Information website (https://blast.ncbi.nlm.nih.gov/Blast.cgi; accessed on 4 August 2023 and repeated on 27 September 2023).

### 3.3. Bacterial Growth

#### 3.3.1. Shaken Flasks

Erlenmeyer flasks with a 250 mL volume containing 40% volume of media were used for cultivation with different media, namely: Mueller–Hinton (MH); Luria–Bertani (LB); Marine broth (MB; Condalab, Madrid, Spain); Terrific broth (TB; 5 g/L glycerol, 12 g/L tryptone, 24 g/L yeast extract, 15 g/L agar, and potassium phosphate buffer 70 mM, pH 7.2); BBL^TM^ Trypticase Soy Broth (TSB; BBL, Sparks, MD, USA); Guillard’s (F/2) medium (F2; Sigma); cyanobacteria BG-11 freshwater solution (BG-11; Sigma); and modified LB medium (RP; 5 g/L of yeast extract, 30 g/L of sodium chloride, 10 g/L of glucose, 10 g/L of meat peptone, as stated in [65]); MH and LB were also tested supplemented with 30 g/L NaCl and with TB without glycerol and 30 g/L NaCl. The flasks were incubated at 30 °C and 150 rpm in an Agitorb 200 incubator (Aralab, Portugal). All assays were performed at least in duplicate.

#### 3.3.2. Microtiter Plates (MTPs) with Oxygen and pH Monitoring

The effect of salinity was determined in RP, TB, and mineral medium (MM; [66]) on 24-well microtiter plates (Sarstedt, Nümbrecht, Germany) containing 1.5 mL per well. To assess the effect of oligotrophic conditions, MB was diluted 5, 10, 15, 20, 35, and 40 times, and the dissolved oxygen concentration was monitored on-line using 24-well OxoDish^®^ plates (from PreSens Precision Sensing GmbH, Regensburg, Germany) containing 1.5 mL of cell culture per well. To study the influence of pH, cells were grown in MB at pH 6.0, 7.2, 7.5, 8.0, and 8.5 using 6-well HydroDish^®^ plates (from PreSens) with 4.5 mL of cell culture per well. Data acquisition was carried out using the software SDR_v37 (also from PreSens).

According to the manufacturer, the Oxodish^®^ microtiter plates have a resolution of ±0.4% O_2_, a precision of ±1% O_2_ at 20.9% O_2_, and a drift <0.2% O_2_ within one week, whilst HydroDish^®^ plates present a measurement range of pH 6–8.5, with a resolution of ±0.05 and accuracy of ±0.1 at pH 7.

The plates were incubated at 30 °C and 150 rpm. In all assays, performed at least in duplicate, biomass and product production were also measured.

### 3.4. Bacterial Adaptation

#### 3.4.1. Zeta Potential

After harvesting the cells using centrifugation (Sorvall RC6, ThermoFisher), these were washed twice with 10 mM of KNO_3_. The pellet was then resuspended in the same solution and, depending on the sample OD_600 nm_, diluted 50 to 100 times. The zeta potential was measured at 25 °C on a Doppler electrophoretic light scattering analyser (Zetasizer Nano ZS from Malvern Instruments, Malvern, UK). The zeta potential was determined from the electrophoretic mobility data with the Zetasizer software 7.10, also from Malvern Instruments. The results are the average of at least two independent cultures grown under each tested condition.

#### 3.4.2. Lipid Composition

To assess changes in the fatty acid composition of the cellular membranes which occur as a response to environmental stresses, the lipid content of the cells was extracted, and the fatty acid composition was determined by gas chromatography. Cells were harvested at the end of the exponential growth phase by harvesting 1 mL of culture medium and washed twice with milli-Q water. The FAs were extracted and simultaneously methylated to FAMEs, using the Instant FAME^TM^ method from MIDI, as described previously [1,20]. FAMES were analysed on the gas chromatograph mentioned in Section 3.2. using the PLFAD1 method. The results are the average of lipid extractions of at least two independent cultures grown under each tested condition.

### 3.5. Product Characterization

#### 3.5.1. Extraction Procedure

To monitor the production along growth, a rapid method was used, as described previously [57]. Briefly, 1 mL samples of cell suspension were taken during cell growth and centrifuged at 12,500× *g* for 8 min. The resulting pellets were resuspended and washed with 1 mL of Milli-Q water. After another centrifugation cycle, the pellets were resuspended in a 3:1 (*v*/*v*) solution of ethyl acetate:acetic acid glacial (both from Sigma-Aldrich, St. Louis, MO, USA) and left protected from light for 1 h at room temperature. After centrifugation at 7000× *g* for 5 min, the supernatants were analysed with UV-VIS spectroscopy between 200 and 800 nm.

After fermentation, the product was extracted from the cells with a modified procedure based on [58]. In summary, the cell culture was centrifuged at 12,000× *g* for 15 min, and the resulting pellet was washed with Milli-Q water. After a new centrifugation cycle, the cells were chemically disrupted with cold isopropanol (Fisher Scientific, Loughborough, UK), and the red prodiginine was left to be extracted overnight. The resulting coloured supernatant was evaporated with a RapidVap Vacuum Evaporation System (Labconco, Kansas City, MO, USA) until dryness. The resulting purple product was resuspended in dichloromethane (Fisher Scientific, Waltham, MA, USA) and washed (30:6, *v*/*v*), first with water followed by a brine solution, until the polar fraction was clear. The organic phase, containing the product, was concentrated until ca. 5 mL and loaded to a chromatographic column containing silica gel 60 (0.015–0.040 mm; Merck, Darmstadt, Germany) as the stationary phase. The equilibrium of the column was carried out with dichloromethane, and the product was eluted with dichloromethane followed by methanol (Fisher Scientific, Waltham, MA, USA). The resulting fractions were pooled according to their spectrophotometric spectra, evaporated until dryness, and the weight of the resulting product was determined.

#### 3.5.2. Thin-Layer Chromatography

The efficacy of the purification procedure was assessed by thin-layer chromatography (TLC) on pre-coated silica gel 60 F254 (Merck, Darmstadt, Germany) aluminium sheets, using a dichloromethane:methanol solution (1:1 *v*/*v*) as eluent. Detection of the band(s) was performed with visual inspection under visible and ultraviolet (254 nm) light. A two-dimensional TLC (2D–TLC) was also used to confirm that no other bands were present in the purified product. In these analyses, dichloromethane was used as the eluent in one direction, followed by methanol in the 90° direction.

### 3.6. Analytical Methods

#### 3.6.1. UV-VIS Spectroscopy

Cellular growth and product production were monitored off-line using UV-VIS spectroscopy at 600 nm and 200–800 nm, respectively, in a Multiskan Go spectrophotometer (Thermo Scientific, Waltham, MA, USA) or in a T80 UV/VIS spectrophotometer (PG Instruments Limited, Leicestershire, UK). The highest peak in the UV-VIS region was observed at 535 nm, which allowed the quantification of the product in ethyl acetate:acetic acid (3:1, *v*/*v*) by using the following calibration curve (Equation (2)):

(2) $$\left[P(mg/L)\right]=32.2{Abs}_{535\mathrm{nm}}(R^{2}=0.999)$$

#### 3.6.2. Fluorescence Microscopy and Spectroscopy

The product dissolved in the ethyl acetate:acetic acid mixture was dispersed over a microscope glass slide, and after the evaporation of the solvents, the solid product was observed using an Olympus CX40 microscope (Olympus, Tokyo, Japan) equipped with an Olympus U-RFL-T burner and mirror cube units, allowing the observation of the crystals under fluorescent light at different wavelengths. Images were captured with an Evolution™ MP5.1 CCD colour camera using the software Image-Pro Plus, both from Media Cybernetics, Inc. (Rockville, MD, USA). The product was extracted from the cells using methanol and chloroform (both from Fisher Scientific, Waltham, MA, USA), and the spectra of both mixtures were analysed in a Fluorolog 3–22 Spectrofluorometer with FluorEssence Software version 3.1.5.11 (both from HORIBA, Ltd., Kyoto, Japan). The emission and excitation wavelengths were set between 400 and 800 nm and between 200 and 600 nm, respectively.

#### 3.6.3. Nuclear Magnetic Resonance (NMR)

The dried purified product was dissolved in 0.5 mL of deuterated chloroform (Eurisotop, Saint-Aubin, France) and transferred to a 5 mm NMR tube. All NMR spectra were recorded on a Bruker 500 MHz Avance II spectrometer (Bruker, Billerica, MA, USA), using either a 5 mm TXI inverse probe or a 5 mm Broad Band probe. ^1^H and ^13^C were observed at 500.13 and 125.76 MHz, respectively. Data acquisition and processing were performed using the TopSpin 4.1 software from Bruker, Billerica, MA, USA.

#### 3.6.4. Elemental Analysis

To determine the elemental composition of the purified product, an elemental analysis for carbon, hydrogen, nitrogen, and sulphur (C, H, N, S) was performed in the accredited laboratory LAIST (Lisboa, Portugal), according to their certified method M.M 8.6 (certification date: 6 May 2009).

#### 3.6.5. Fourier-Transform Infrared Spectroscopy-Attenuated Total Reflectance (FTIR-ATR)

The FTIR-ATR spectrum of the dried purified product was measured with a Nicolet 5700 FTIR (Thermo Scientific, Waltham, MA, USA) at a wavelength between 555 and 4000 cm^−1^ with a resolution of 1 cm^−1^.

#### 3.6.6. Differential Scanning Calorimetry (DSC)

The purified product was diluted in dichloromethane, placed in a high-pressure aluminium crucible, and allowed to dry at room temperature until ≈ 1 mg of product/crucible. The resulting thermograms were performed with DSC 200 F3 Maia equipment (NETZSCH, Bayern, Germany) using a heating/cooling rate of 10 °C/min, between 20 °C and 300 °C. An empty crucible was used in the reference cell. The thermograms were analysed using the software NETZSCH Proteus Thermal Analysis.

#### 3.6.7. Gas Chromatography-Mass Spectrometry (GC-MS)

The dried product was dissolved in methyl *tert*-butyl ether (MTBE; Sigma-Aldrich, Waltham, MA, USA) and analysed on an Agilent 7820A gas chromatograph equipped with a 7693A autoinjector and an Agilent 5977E quadrupole MS detector (all from Agilent Technologies, Santa Clara, CA, USA). The capillary column used was an Agilent J&W Ultra 2, working at a constant flow of 1 mL/min. The GC injector was set at 200 °C, the MS source at 230 °C, the MS quad at 150 °C, and the MSD transfer line at 280 °C. The separation of products was achieved by programming the oven to an initial temperature of 50 °C and increasing the temperature to 220 °C at 4 °C/min, followed by a second increase to 280 °C at 10 °C/min. Peak identification was performed using the Qualitative Analysis B.07.00 software, part of the MassHunter Workstation from Agilent, by comparison to the NIST mass spectral library version 2.2.

#### 3.6.8. Ultra-High Resolution Qq Time-of-Flight Mass Spectrometry

The purified product was diluted in acetonitrile (Fisher Scientific, Waltham, MA, USA) and analysed by direct infusion on a QqTOF Impact II^TM^ mass spectrometer (Bruker Daltonics, Bremen, Germany) operating in the ESI positive mode. Internal calibration was achieved with a solution of ammonium formate 10 mM, and the full-scan mass spectra were acquired between a mass range of 100–1000 *m*/*z*, at a spectra rate of 1 Hz. The HR tandem mass spectrum was acquired in the MRM mode with a collision energy of 25 eV. Data acquisition and processing were performed using the Data Analysis 4.2 software (Bruker Daltonics, Bremen, Germany).

## 4. Conclusions

The *S. rubidaea* isolated from a marine sample collected near a thermal vent presented a significantly wide biotic window, being able to grow and to produce prodigiosin from 15 to 62 °C, under low nutrient conditions, and at different pH values. Nevertheless, the environmental conditions and media composition influenced both the biomass and product production, with the cells being able to modulate the fluidity of the cellular membrane.

To make an accurate identification of the chemical structure of the red product produced, several techniques were used for its extensive physico-chemical characterization. It was concluded that the product produced by *S. rubidaea* has a prodigiosin moiety. However, its full structure, specifically in the aliphatic region, remains undetermined due to the presence of an aliphatic molecule, detected by NMR, that remained bonded to the aliphatic region of prodigiosin, regardless of the purification technique tested. It is hypothesized that the production of this aliphatic molecule is a survival mechanism for the strain to retain prodigiosin within reach, as it could act as an anchor for the molecule.

## Figures and Tables

**Table 1:** Fatty acid composition of *S. rubidaea* cells when grown at 15 to 62 °C in MB.

Fatty Acid (%)	Temperature (°C)				
	15	20	30	37	62
12:0	0.23	0.26	0.31	0.54	8.80
14:0	1.53	2.22	3.58	5.21	0.00
15:0	0.35	1.03	1.21	1.17	0.00
15:3 ω3c	0.09	0.08	0.13	0.15	3.87
16:0	25.73	28.39	36.66	40.85	14.85
16:1 ω5c	0.29	0.24	0.14	0.11	0.00
16:1 ω7c	33.43	29.48	13.16	3.35	4.66
16:4 ω3c	2.12	1.60	1.57	1.42	1.47
17:0	0.23	0.42	0.51	0.63	0.00
17:0 *cyclo* ω7c	5.72	7.42	23.39	32.06	1.34
18:0	0.38	0.91	0.68	0.86	14.05
18:1 ω7c	28.56	26.50	16.03	7.20	7.44
18:1 ω9c	0.00	0.08	0.35	0.11	1.29
18:3 ω6c	1.05	0.91	1.44	1.71	42.23
19:0 *cyclo* ω7c	0.27	0.44	0.84	4.61	0.00

**Table 2:** Sample location and environmental conditions.

Location	GPS Coordinates	T_H_2_O_ (°)	T_air_ (°)	pH	Conductivity (mS/cm)
S. Miguel, Azores	37°51′30″ N 25°51′7″ W	28	18	8.91	66.6

## Data Availability

The data presented in this study are available on request from the corresponding author.

## Supplementary Materials

The following supporting information can be downloaded at https://www.mdpi.com/article/10.3390/md21120599/s1, Table S1: NMR experimental data used in the structural elucidation of the putative prodigiosin. Q—quaternary; T—tertiary; S—secondary; P—primary.
