# Supplementary material for: Isolation and Characterization of a Serratia rubidaea from a Shallow Water Hydrothermal Vent
Source: Mar Drugs. 2023 Nov 21;21(12):599. doi: 10.3390/md21120599 (PMC10745058; doi:10.3390/md21120599)
Supplement: Supplementary file 1 [file marinedrugs-21-00599-s001.zip › marinedrugs-2721576-supplementary.pdf]

Isolation and characterization of a Serratia rubidaea from a shallow water hydrothermal vent

Ricardo F. S. Pereira [1,2], Maria J. Ferreira[3], M. Conceição Oliveira[3], Maria C. Serra[4], Carla C. C. R. de Carvalho [1,2*]

[1] iBB – Institute for Bioengineering and Biosciences, Department of Bioengineering, Instituto Superior Técnico, Universidade de Lisboa, Av. Rovisco Pais, 1049-001 Lisboa, Portugal; ricardofspereira@tecnico.ulisboa.pt
[2] Associate Laboratory i4HB – Institute for Health and Bioeconomy, Instituto Superior Técnico, Universidade de Lisboa, Av. Rovisco Pais, 1049-001 Lisboa, Portugal
[3] Centro de Química Estrutural, Institute of Molecular Sciences, Instituto Superior Técnico, Universidade de Lisboa, 1049-001 Lisboa, Portugal; m.joao.ferreira@tecnico.ulisboa.pt; conceicao.oliveira@tecnico.ulisboa.pt
[4] Área Departamental de Engenharia Química, Instituto Superior de Engenharia de Lisboa (ISEL), Rua Conselheiro Emídio Navarro, 1, 1959-007 Lisboa, Portugal; mcserra@deq.isel.ipl.pt
* Correspondence: ccarvalho@tecnico.ulisboa.pt; Tel.: +351 21 841 95 94

Supplementary Information

Table S1. NMR experiments data used in the structural elucidation of the putative prodigiosin. Q – quaternary; T – terciary, S – secondary; P – primary.

	^{1}H δ	^{1}H Integration	^{13}C Jmod δ	^{1}H-^{13}C HMBC	^{1}H-^{1}H COSY	^{1}H-^{1}H NOESY
**1**	12.59	1H	-	-	3, 4, 5	5
**2**	-	-	122.2 (Q)	2, 4	-	-
**3**	6.94	1H	117.0 (T)	3, 4, 5	1, 4	4, 4’
**4**	6.37	1H	111.9 (T)	2, 3, 5	1, 3, 5	3, 5
**5**	7.25	1H	127.0 (T)	2, 3, 4	1, 4	1, 4
**1’**	12.70	2H	-	2’, 3’, 4’, 5’,8’	4’, 8’, 11’’	1, 11’’
**2’**	-	-	120.9 (Q)	1’, 4’	-	-
**3’**	-	-	165.9 (Q)	1’, 7’, 4’’	-	-
**4’**	6.10	1H	92.9 (S)	2’, 5’	1’	3, 7’
**5’**	-	-	147.5 (Q)	4’, 8’ 11’’	-	-
**7’**	4.03	3H	58.9 (OCH_3)	3’	-	4’
**8’**	6.70	1H	128.3 (T)	2’’, 4’’	1’, 11’’	4’’
**3’’**	-	-	146.9 (Q)	8’, 11’’	-	-
**4’’**	6.97	1H	116.0 (T)	3’, 8’	8’	8’
**6’’**	2.43-2.34	4H	33.7 (S)	12’’	7’’	7’’
**7’’**	1.70-1.52	4H	29.9 (S)	8’’	9’’, 10’’	6’’, 9’’
**8’’**	1.33	7 H	22.4 (S)	-	7’’, 10’’	7’’
**9’’**	1.22	15H	29.6 (S)	7’’	7’’, 10’’	-
**10’’**	0.93	6H	14.2 (P)	9’’	8’’	8’’
**11’’**	2.57	3H	12.7 (P)	8’, 2’’	1’, 8’	1’
